# Genome-Wide Analysis of the *Wall-Associated Kinase* (*WAK*) Genes in *Medicago truncatula* and Functional Characterization of *MtWAK24* in Response to Pathogen Infection

**DOI:** 10.3390/plants12091849

**Published:** 2023-04-30

**Authors:** Weiyi Kong, Jia Shi, Bo Yang, Shuhan Yu, Pengcheng Zhao, Zhenfei Guo, Haifeng Zhu

**Affiliations:** 1 College of Grassland Science, Nanjing Agricultural University, Nanjing 210095, China; kongweiyi@njau.edu.cn (W.K.); 2021120018@stu.njau.edu.cn (J.S.); yangb@njau.edu.cn (B.Y.); 2018220001@njau.edu.cn (S.Y.); 2016220006@njau.edu.cn (P.Z.); 2 Jiangsu Key Laboratory for the Research and Utilization of Plant Resources, Institute of Botany, Jiangsu Province and Chinese Academy of Sciences (Nanjing Botanical Garden Mem. Sun Yat-Sen), Nanjing 210014, China

**Keywords:** abiotic stress, biotic stress, expression pattern, *Medicago truncatula*, WAK

## Abstract

The wall-associated kinases (WAKs) can perceive and transmit extracellular signals as one kind of unique receptor-like kinases (RLKs) involved in the regulation of cell expansion, pathogen resistance and abiotic stress tolerance. To understand their potential roles and screen some key candidates in *Medicago truncatula* (*M. truncatula*), genome-wide identification and characterization of *MtWAKs* were conducted in this study. A total of 54 *MtWAK* genes were identified and classified into four groups based on their protein domains. They were distributed on all chromosomes, while most of them were clustered on chromosome 1 and 3. The synteny analysis showed that 11 orthologous pairs were identified between *M. truncatula* and *Arabidopsis thaliana* (*A*. *thaliana*) and 31 pairs between *M. truncatula* and *Glycine max* (*G*. *max*). The phylogenetic analysis showed that WAK-RLKs were classified into five clades, and they exhibited a species-specific expansion. Most *MtWAK-RLKs* had similar exon–intron organization and motif distribution. Multiple *cis*-acting elements responsive to phytohormones, stresses, growth and development were observed in the promoter regions of *MtWAK-RLKs*. In addition, the expression patterns of *MtWAK-RLKs* varied with different plant tissues, developmental stages and biotic and abiotic stresses. Interestingly, plasm membrane localized MtWAK24 significantly inhibited *Phytophthora* infection in tobacco. The study provides valuable information for characterizing the molecular functions of *MtWAKs* in regulation of plant growth, development and stress tolerance in legume plants.

## 1. Introduction

Plants perceive and process various signals on cell surface to modulate biological processes through members of the receptor-like kinase family [1]. WAK is a unique class of RLKs that link cell wall to cytoplasm physically, consisting of extracellular epidermal growth factor (EGF) domain and/or galacturonan-binding (GUB) domain, transmembrane domain and intercellular Ser/Thr kinase domain [2]. Based on the organization of the conserved domains, WAKs are divided into four types: the RLK type, which contains both extracellular domain and kinase domain; the receptor-like cytoplasmic kinase (RLCK), which contains kinase domain alone; the receptor-like protein (RLP), which contains only extracellular domain; and the short protein type, which harbors no domain but shows high similarity in the emerging sequence.

WAKs participate in regulation on plant growth and development as well as biotic and abiotic stress tolerance. Down-regulation of *AtWAK4* or *AtWAK2* expression leads to impaired cell expansion in *Arabidopsis* [3,4]. OsWAK11 regulates both stem and seed elongation in rice through monitoring cell wall pectin changes that fine-tune brassinosteroid signaling [5]. OsiWAK1 positively regulates male sterility by binding its extracellular domain to pectic polysaccharides and plays a pivotal role in regulating programmed cell death process [6]. Several *WAK* genes, such as *AtWAK1* [7], *Htn1* (*ZmWAK*) [8,9], *Stb6* [10] and *Xa4* [11], are involved in plant immunity against fungal and bacterial pathogens. *OsWAK14*, *OsWAK91* and *OsWAK92* expression is up-regulated by inoculation with *Magnaporthe oryzae* and chitin treatment for regulating blast resistance in rice positively. On the contrary, *OsWAK112d* expression is down-regulated by *M. oryzae* and negatively regulates blast resistance [12]. The *AtWAK1* transcript level is induced by oligogalacturonides (OGs) that act as damage-associated molecular patterns (DAMPs) for activating plant immune responses [13], and the cytoplasmic kinase domain is activated by OGs [3]. *AtWAK1* [14], *AtWAKL4* [15] and *OsWAK11* [16] are involved in metal tolerance. *AtWAKL4* expression is induced by Na^+^, K^+^, Cu^2+^, Ni^2+^ and Zn^2+^, and the T-DNA insertion of *AtWAKL4* resulted in hypersensitivity to excessive K^+^, Na^+^, Cu^2+^, and Zn^2+^ [15]. OsWAK112 negatively regulates plant salt responses by binding with OsSAMS1/2/3 for inhibiting ethylene production [17].

*M. truncatula* is a diploid plant with a small genome size. It is a model legume species for functional genomics research. However, there are no investigations on WAKs in *M. truncatula*. Given the important role of WAKs in plants, the genome-wide analysis of *WAK* family genes was conducted to provide valuable information for understanding the roles of *MtWAKs* in the model legume in the present study. The chromosomal location, gene duplication and coding protein properties of *MtWAK* genes were analyzed. To highlight the putative functions of the classical RLK type (MtWAK-RLKs), their phylogenetic relationship, gene structure and conserved domains were characterized. Furthermore, their *cis*-acting elements in the promoter and expression profiles in response to diverse stresses were examined.

## 2. Results

### 2.1. Identification and Characterization of WAKs in M. truncatula

To comprehensively identify *WAK* genes in the genome of *M. truncatula*, the data harvested from BLASTP and HMMER searches analysis were integrated. The putative candidates were further verified for the presence of the EGF_CA (PF07645), WAK_assoc (PF14380), GUB_WAK_bind (PF13947) and Pkinase_Tyr (PF07714) domain. A total of 54 non-redundant and full-length *MtWAKs* were obtained (Table S1). According to the classification of *OsWAKs* [18], *MtWAKs* were divided into 4 groups that contained 26 *MtWAK-RLKs*, 9 *MtWAK-RLCKs*, 10 *MtWAK-RLPs* and 9 *MtWAK* short proteins (Table S1). The numbers of amino acid (aa) ranged from 52 (MtWAK32) to 771 (MtWAK29), while the molecular weight (MW) varied from 5.55 kDa (MtWAK32) to 85.6 kDa (MtWAK29). The isoelectric point (pI) of MtWAKs ranged from 4.47 (MtWAK54) to 10.17 (MtWAK32), with an average of 6.94 (Table S1).

### 2.2. Chromosomal Location and Expansion Analysis of MtWAKs

**Figure 1:**
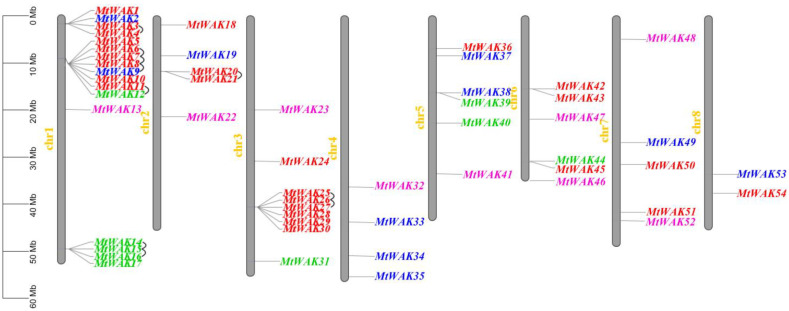
Chromosomal location of *MtWAK*s. Grey bars represent the chromosomes. The chromosome numbers are shown in yellow. The scale bar is shown on the left. WAK gene names are highlighted with different colors. *MtWAK-RLKs, MtWAK-RLCKs*, *MtWAK-RLPs* and *MtWAK* short proteins are indicated using red, green, blue, and pink, respectively. Tandem duplicated gene pairs are linked using black lines.

Fifty-four *MtWAKs* were unevenly distributed on eight chromosomes (Figure 1). Chromosome 1 had 17 *MtWAKs*, while the other chromosomes had 2 to 9 *MtWAKs*. Interestingly, 20 *MtWAK-RLKs* and 7 *MtWAK-RLCKs* were found in clusters with 10 pairs of tandem duplicated gene pairs, while most *MtWAK-RLPs* and *MtWAK* short proteins were separately distributed on 8 chromosomes (Figure 1, Table S2).

**Figure 2:**
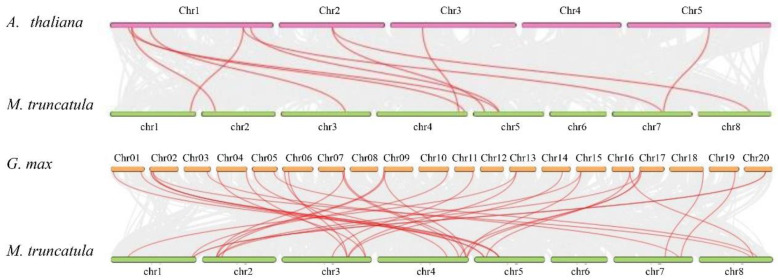
The synteny analysis of *MtWAK*s between *M. truncatula* and *A. thaliana* or *G. max*. Gray lines indicate all collinear blocks within *M. truncatula* and *A. thaliana* or *G. max*, while red lines indicate the synteny of *WAK* genes between *M. truncatula* and *A. thaliana* or *G. max*.

Two pairs of paralogous genes, *MtWAK19*-*MtWAK35* and *MtWAK36*-*MtWAK53*, belong to segmental duplications (Table S2). The synteny analysis showed that there were 11 pairs of orthologous genes between *M. truncatula* and *A. thaliana* and 31 pairs between *M. truncatula* and *G. max* (Figure 2, Table S2).

### 2.3. Phylogeny, Gene Structure, Protein Domain and Motif Analysis of MtWAK-RLK Members

**Figure 3:**
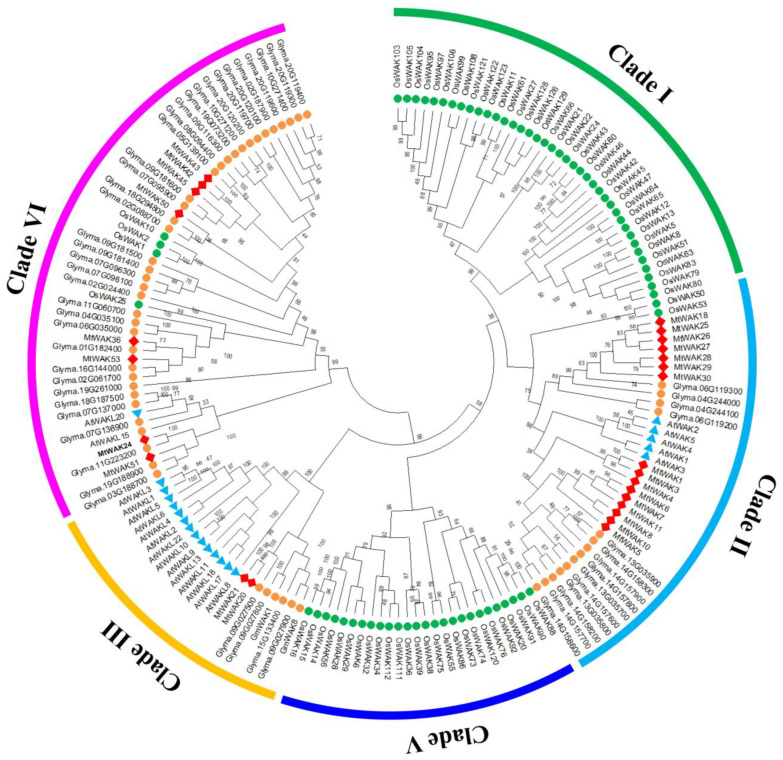
Phylogenetic analysis of WAK-RLKs from *M. truncatula, A. thaliana, G. max* and *O. sativa:* 26 MtWAK-RLKs (red square), 21 AtWAK-RLKs (blue triangle), 56 GmWAK-RLKs (orange circle) and 71 OsWAK-RLKs (green circle) were aligned using the CLUSTALX software (Version 1.81). The phylogenetic tree was constructed with MEGAX software (version 10.0.5) by the neighbor-joining methodwith 1000 bootstrap replicates.

Phylogenetic analysis of WAK-RLKs from *M. truncatula*, *A. thaliana*, *G. max* and *O. sativa* showed that the WAK-RLKs could be divided into five clades. Clade Ⅰ and Ⅴ contained only OsWAK-RLKs. Clade Ⅱ contained 5 AtWAKs, 14 GmWAK-RLKs, 16 MtWAK-RLKs and 2 OsWAK-RLKs. Clade Ⅲ contained 14 AtWAKL-RLKs, 6 GmWAK-RLKs and 2 MtWAK-RLKs. Clade Ⅳ was comprised of 2 AtWAKL-RLKs, 35 GmWAK-RLKs, 4 OsWAK-RLKs and 8 MtWAK-RLKs (Figure 3).

**Figure 4:**
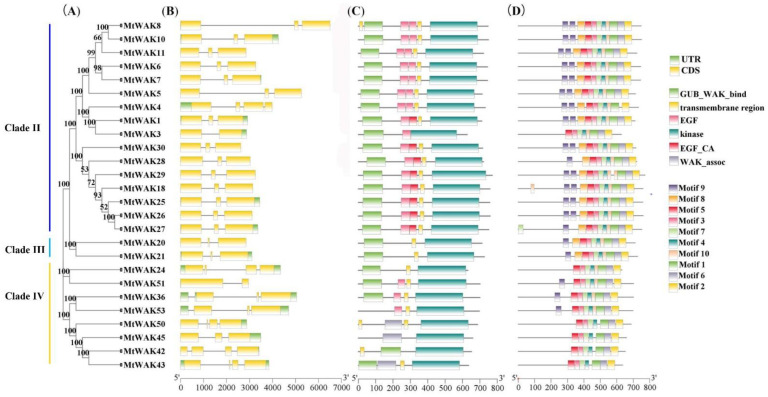
The gene structure, conserved domain and motif analysis of MtWAK-RLKs. (**A**) The phylogenetic tree of MtWAK-RLKs. (**B**) The gene structure of *MtWAK-RLKs*. (**C**) The conserved domain of MtWAK-RLKs. (**D**) The top ten conserved motifs of MtWAK-RLKs.

The gene structure, protein domain and motif of MtWAK-RLKs were further analyzed (Figure 4 and Figure S1). Most members showed similar gene structure, harboring two long exons at both ends and a short one in the middle, except *MtWAK3* and *MtWAK51* with two exons only and *MtWAK4*, *24*, *42*, *43* and *50* with four exons (Figure 4B). One or two transmembrane domains were shown in the majority of MtWAK-RLKs except MtWAK3 and MtWAK45. Both Gub_WAK_bind and EGF domains existed in all members of clade Ⅱ and MtWAK36, and there were 51 in clade Ⅳ (Figure 4C). Moreover, 10 conserved motifs were predicted using MEME program. Motifs 1 to 7 corresponded to the conserved kinase domain in all MtWAK-RLKs (Figure 4D and Figure S1). A conserved arginine (R) residue was present in front of the motif DxxxxN; thus, all MtWAK-RLKs were classified as RD kinase. In addition, motif 8 was absent in the members of clade Ⅳ.

### 2.4. Analysis of Cis-Acting Elements in the Promoter Region of MtWAK-RLKs

**Figure 5:**
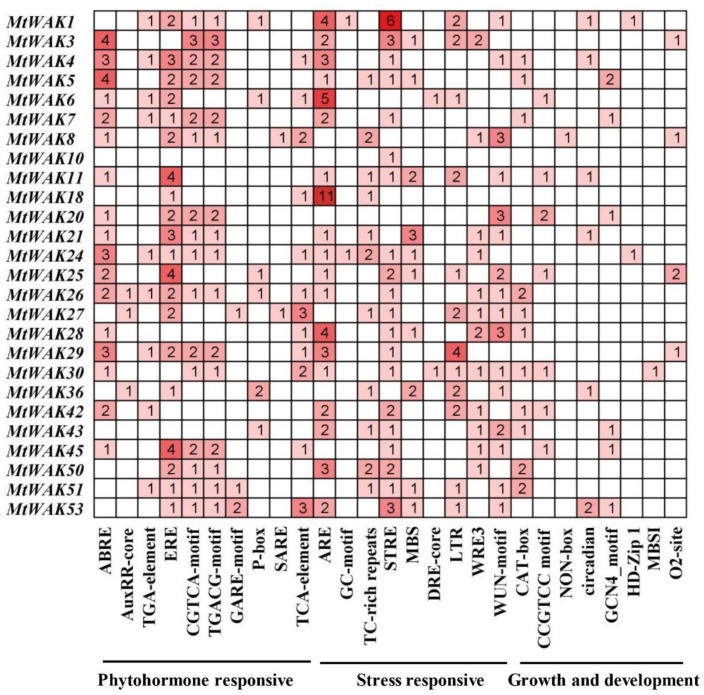
*Cis*-acting elements in the promoters of *MtWAK-RLK*s. The numbers and the depth of red represent the frequency of the elements. ABRE, ABA-responsive element; AuxRR-core and TGA-element, auxin-responsive elements; ERE, ethylene-responsive element; CGTCA and TGACG motif, MeJA-responsive elements; SARE and TCA-element, salicylic-acid-responsive elements; GARE-motif and *P*-box, GA-responsive elements; ARE and GC-motif, anaerobic induction elements; TC-rich repeats and STRE, stress-responsive elements; MBS, drought-induced element; DRE-core, dehydration-responsive element; LTR, low-temperature-responsive element; WRE3 and WUN-motif, wound-responsive element; CAT-box, meristem expression element; CCGTCC motif and NON-box, meristem-specific activation elements; circadian, circadian control element; GCN4_motif, endosperm-expression element; HD-Zip 1, palisade mesophyll cell-expression element; MBS, flavonoid biosynthetic gene regulation element; O2-site, zein metabolism regulation element.

To understand the potential function of *MtWAK-RLKs*, the putative *cis*-acting elements were analyzed using PlantCARE software. Abundant phytohormone and stress responsive elements were observed in the promoter regions (Figure 5). Twenty-four *MtWAK-RLKs* possessed a larger number of ABA-responsive elements, ethylene-responsive elements (ERE) and MeJA responsive elements (CGTCA-motif, TGACG-motif). Twelve *MtWAK-RLKs* had salicylic-acid-responsive elements (TCA-element), while nine *MtWAK-RLKs* had auxin-responsive elements (TGA-element). Most *MtWAK-RLKs* had anaerobic induction elements (ARE) and stress-responsive elements (STRE) in the promoter region (Figure 5). A series of growth and development related *cis*-elements, such as meristem-expression element (CAT-box) and CCGTCC motif were observed in *MtWAK-RLKs*. The results suggested that MtWAK*-RLK*s might be involved in phytohormone regulation and stress response in *M. truncatula*.

### 2.5. Expression Analysis of MtWAK-RLKs across Different Tissues and Developmental Stages

**Figure 6:**
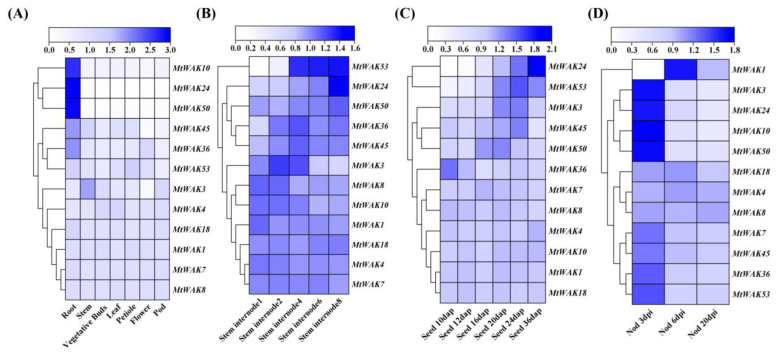
Clustered heatmap of the relative expression levels of *MtWAK-RLK*s in different tissues and developmental stages. (**A**) Different organs. (**B**) Developing seeds. (**C**) Developing internodes. (**D**) Developing nodules. Microarray data are normalized by the mean expression value of each gene in tissues. The clustered heatmap on log2 scale is drawn using TBtools software (Version 1.108). DAP, days after pollination. DPI, days post-incubation.

To obtain insights into their temporal and spatial expression patterns, 12 *MtWAK-RLKs* having corresponding probesets in the gene expression database were prioritized for analysis (Table S3). *MtWAK1*, *4*, *7*, *8* and *18* were almost equally expressed in all tissues (Figure 6A). *MtWAK10*, *24* and *50* were exclusively and highly expressed in roots. *MtWAK36* and *45* were highly expressed in roots, whereas *MtWAK3* was highly expressed in stem. From base to apex of stem, *MtWAK24* and *MtWAK53* expression was increased gradually, while *MtWAK3* exhibited the opposite trend with the lowest expression level in the top internode (Figure 6B). Interestingly, *MtWAK24* and *53* were significantly up-regulated along with seed development (Figure 6C). In addition, most *MtWAK-RLK*s were down-regulated during nodulation, except that *MtWAK1* was up-regulated greatly at 6 dpi and 20 dpi (Figure 6D).

### 2.6. Expression Analysis of MtWAK-RLKs in Response to Biotic and Abiotic Stresses

**Figure 7:**
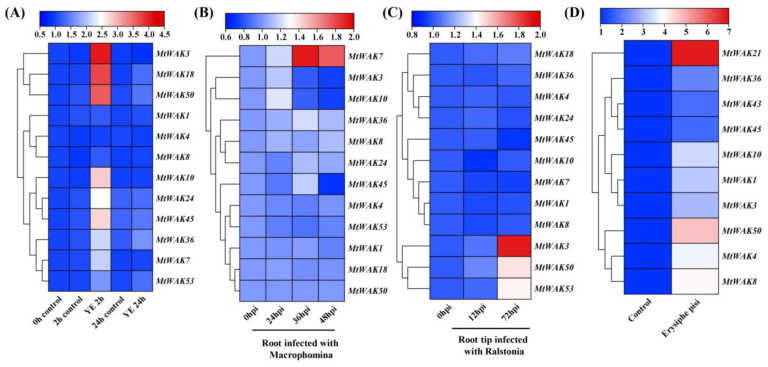
Clustered heatmap of the relative expression levels of *MtWAK-RLK*s involved in biotic stress response. (**A**) Yeast eliciter (YE) treatment. (**B**) *Macrophomina* infection. (**C**) *Ralstonia* infection. **(D**) *Erysiphe pisi* infection. The relative gene expression levels after treatments were calculated and compared with the untreated control or before treatment. The clustered heatmap on log2 scale was drawn using TBtools software (Version 1.108).

To understand the expression of *MtWAK-RLKs* in response to pathogens, datasets of ‘Cell suspension_Yeast elicitor’, ‘Root_*Macrophomina* infected’ and ‘Root Tip_A17_*Ralstonia*’ from the MtGEA were used for analysis (Table S3). Transcripts of nine *MtWAK-RLKs* were induced greatly after 2 h of treatment with yeast elicitor (Figure 7A). Although most *MtWAK-RLKs* were not responsive to infection by *Macrophomina* or *Ralstonia*, *MtWAK7* transcript was induced greatly in roots at 36 and 48 hpi with *Macrophomina* (Figure 7B), and *MtWAK3*, *50* and *53* transcripts were up-regulated at 72 hpi with *Ralstonia* (Figure 7C). In addition, seven *MtWAK-RLK*s were up-regulated by infection with *Erysiphe pisi* (Figure 7D).

**Figure 8:**
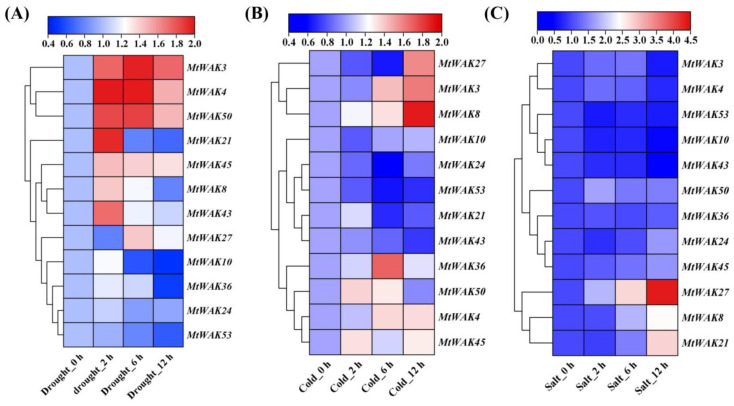
Clustered heatmap of the relative expression levels of *MtWAK-RLK*s involved in abiotic stress response. (**A**) Drought stress. (**B**). Cold stress (**C**) Salt stress. The relative gene expression levels after treatments were calculated and compared with the control untreated or before treatment. The clustered heatmap on log2 scale was drawn using TBtools software (Version 1.108).

*MtWAK-RLKs* were responsive to abiotic stress (Figure 8, Table S3). *MtWAK8*, *21* and *43* were up-regulated at 2 h after drought stress, while *MtWAK27* was down-regulated. *MtWAK2*, *5*, *45* and *50* were continuously up-regulated after 2 h of drought stress, and *MtWAK24* was not responsive to drought (Figure 8A). *MtWAK21*, *24*, *43* and *53* were down-regulated in response to cold stress*,* while *MtWAK10* was not responsive to cold. *MtWAK3*, *4*, *8*, *27*, *36*, *45* and *50* were up-regulated after cold treatment (Figure 8B). *MtWAK8*, *21* and *27* were up-regulated after 12 h of salt treatment, while *MtWAK3*, *10*, *43* and *54* were down-regulated (Figure 8C).

### 2.7. Gene Expression Validation of MtWAK-RLKs by qRT-PCR 

**Figure 9:**
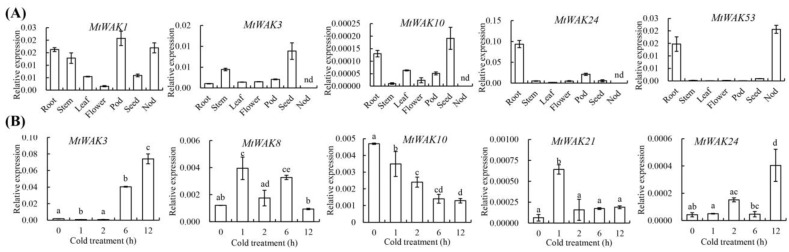
Expression analysis of selected *MtWAK-RLK* genes in different tissues and under cold stress using qRT-PCR. (**A**) Different tissues. (**B**) Cold stress. The error bars were obtained from three measurements. nd indicates not detected. Significant differences are indicated as different lowercase letters (*P* ≤ 0.05, by one-way ANOVA).

To verify the expression profiles obtained from microarray data, transcripts of five *MtWAK*-RLK*s* (*MtWAK1*, *3*, *10*, *24*, and *53*) in different tissues, including root, stem, leaf, flower, pod and seed, were detected using qRT-PCR. The data showed the expression patterns in different tissues were in consistence with the microarray data. For example, *MtWAK10*, *24* and *53* were highly expressed in roots (Figure 9A). The response of transcript levels in five *MtWAK*-RLK*s* (*MtWAK3*, *8*, *10*, *21*, and *24*) were also detected using qRT-PCR. *MtWAK3*, *8* and *21* were up-regulated after cold treatment, while *MtWAK10* was down-regulated (Figure 9B), which was consistent with the microarray data.

### 2.8. Plasma Membrane Localized MtWAK24 Inhibited Phytophthora Infection in Tobacco

**Figure 10:**
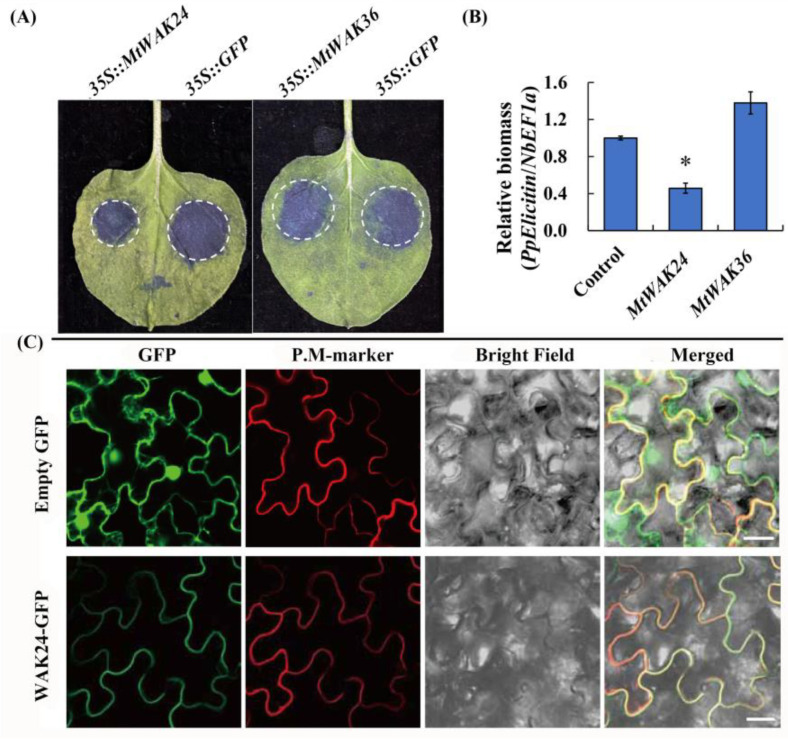
Plasma membrane localized MtWAK24 inhibited Phytophthora infection in tobacco. *N. benthamiana* leaves individually expressing *35S::MtWAK36*, *35S::MtWAK24* or *35S::GFP* were inoculated with *P. parasitica* mycelial plugs at 24 h after *Agrobacterium* infiltration. At 48 hpi, infected leaves were stained using Evans blue staining for lesion determination (**A**). Relative biomass of *P. parasitica* was determined by qPCR of *P. parasitica* genome DNA normalized to tobacco genome DNA (**B**). The results are indicated with means ± SE, n = 6. Asterisks indicate significant differences (* *p* < 0.05; Student’s *t* test). The subcellular localization of MtWAK24-GFP proteins and a free GFP protein in tobacco (**C**). P. M-marker: a plasma membrane localization protein AtAKT1. Bars = 20 µm.

To explore the functions of some candidate *MtWAKs* in modulating plant immunity, we performed *P. parasitica* infection experiments in *N. benthamiana*. The lesion diameter, as well as relative biomass of *P. parasitica*, in leaves expressing *MtWAK24*, but not *MtWAK36,* was significantly decreased compared to the control leaves expressing empty vector, indicating that *MtWAK24* could inhibit the pathogen infection by *P. parasitica* (Figure 10A,B).

Subcellular localization of *MtWAK24* was further analyzed. The data showed that GFP protein was located in the cytoplasm and nucleus, while MtWAK24 protein was located in plasma membrane because the fluorescence was overlapped with that of AtAKT1, the plasma membrane marker protein (Figure 10C).

## 3. Discussion

The *WAK* gene family consists of a large number of members. The numbers of WAK proteins identified by the iTAK (http://bioinfo.bti.cornell.edu/tool/itak) program [19] enlarged from moss to dicot and monocot species, indicating a large expansion of *WAK* family genes during the evolutionary process (Table S4). There are 26, 29, 29, 96, 91, 125, 175, 27 and 320 *WAK* genes in the genomes of *Araidopsis* [20], cotton [21], tomato [22], Chinese cabbage [23], barley [24], rice [25], *Populus* [26], *Juglans regia* [27] and *Triticum aestivum* [28], respectively. Through BLASTP and conserved domain searches, 54 *MtWAK* genes were identified in *M. truncatula*, including 26 *MtWAK-RLK*, 9 *MtWAK-RLCK*, 10 *MtWAK-RLP* and 9 short protein types (Table S1). The majority of *MtWAK-RLK* and *MtWAK-RLCK* genes were clustered in chromosome 1 and 3, attributed mainly to tandem duplications, and the rest were distributed separately among chromosomes (Figure 1, Table S2). Compared to 11 pairs of orthologous *WAKs* between *Arabidopsis* and *M. truncatula*, 31 pairs between *M. truncatula* and *G. max* were found, indicating that the expansion of *MtWAK* family might appear before the separation of *G. max* (Figure 2). Phylogenetic analysis showed that *MtWAK25* and *MtWAK26* were close to *AtWAKs* (Figure 3), and *AtWAK4-MtWAK25* and *MtWAK25-MtWAK26* were orthologous and paralogous pairs, respectively (Table S2), indicating that *MtWAK25* and *MtWAK26* were probably involved in cell expansion like *AtWAK4* [3].

Multiple *cis*-acting elements responsive to phytohormones, stresses, growth and development were observed in the promoter regions of *MtWAK-RLKs*, indicating their potential roles in these processes. The *GhWAKs/WAKLs* having the above *cis*-elements in the promoter were responsive to multiple phytohormones and abiotic stresses [12]. Transcript levels of five *TaWAKs* in wheat (*Triticum aestivum*) were altered by treatments with GA, BR, IAA, JA and ABA [29]. Gene function is associated with its tissue specific expression pattern. *MtWAK10*, *24* and *50* showed a root-specific expression with extremely low expression in other organs. *MtWAK36* and *MtWAK45* were also mainly expressed in roots, whereas *MtWAK3* and *MtWAK53* were highly expressed in stem and petiole, respectively. *MtWAK1*, *4*, *7*, *8* and *18* were evenly expressed in each organ, but the transcript levels were lower than that of other genes (Figure 6A, Table S3). Diverse tissue expression patterns of *MtWAK-RLKs* implied that they might function broadly in plant tissues.

Plant cell expansion and elongation depend on turgor maintenance and cell wall modification, which is associated with the rigidity and elasticity of the cell wall [30]. *WAK* genes can monitor pectin and participate in both turgor maintenance and cell wall modification [31]. *AtWAK4* and *AtWAK2* positively regulate cell expansion [3,4], *HvWAK1* positively regulates root growth [32], while *OsWAK11* regulates both stem and seed elongation [5]. Transcript levels of *MtWAK24* and *MtWAK53* were increased gradually from bottom to top internodes and along with seed growth, while expressions of *MtWAK3* and *MtWAK45* increased, followed by a decrease from internode 1 to internode 8 and along with seed development (Figure 6B,C, Table S3), indicating that they are probably involved in the regulation of cell elongation. 

Cell wall modification is involved in symbiosis between rhizobia and legume plants. Passing of an infection thread from cell to cell requires local cell wall degradation [33]. Modifications in the localization of high- and low-methylated homogalacturonans were detected in nodules [34]. The majority of *MtWAK-RLKs* were greatly down-regulated in the process of nodulation, while *MtWAK1* transcript was increased dramatically, implying that *MtWAKs* are probably associated with nodulation in leguminous plants (Figure 6D, Table S3).

A large body of investigations revealed that *WAK* genes regulate pathogen resistance. *AtWAK1* [7], *AtWAKL22* [35], *Htn1* (ZmWAK) [8,9], *Stb6* [10], *Xa4* [11], *OsWAK1* [36], *OsWAK90-92* [37], *OsWAK25* [38], *SiWAK1* [39], *TaWAK6* [40], *GhWAK7A* [41], *CsWAKL08* [42] and *Rlm4/7/9* [43] positively regulate resistance to various pathogens, and *OsWAK112d* and *Sbs1/2* negatively regulate the defense against pathogenic fungus [12,44]. *MtWAK3*, *18*, *50*, *10*, *24*, *45*, *36*, *7* and *53* transcripts were rapidly induced by yeast elicitor treatment (Figure 7A). Among them, *MtWAK7* was up-regulated by *Macrophomina* infection other than *Ralstonia*, and *MtWAK3*, *50* and *53* were up-regulated by *Ralstonia* other than *Macrophomina* infection (Figure 7B, C), indicating that these genes had specific roles in resistant to *Macrophomina* or *Ralstonia*. Powdery mildews caused by *Erysiphe pisi* are a serious disease that leads to great decreases in crop production worldwide [45]. Ten *MtWAK-RLK* genes were up-regulated greatly after incubation with *Erysiphe pisi*, indicating that they were probably involved in powdery mildew resistance (Figure 7D). To explore the functions of *MtWAK-RLKs* in plant immunity, we selected two yeast elicitor induced *MtWAKs, MtWAK24* and *36,* for *P. parasitica* infection experiments in *N. benthamiana*. It was revealed that expressing *MtWAK24*, but not *MtWAK36*, inhibited infection by *P. parasitica* (Figure 10).

*WAK* genes are involved in metal, salt, drought and cold tolerance, although the mechanisms remain unknown. OsWAK112 negatively regulates plant salt tolerance, possibly via direct binding with OsSAMS1/2/3 [17]. The *Slwak1* null mutant exhibited disturbed osmotic homeostasis and source-sink balance under long term salinity and thereby reduced fruit yield [46]. The CpGRP1-CpWAK1 complex regulates dehydration-induced morphological changes in *Craterostigma plantagineum* [47]. Twelve *MtWAK-RLK* genes were quickly responsive to drought, salt and cold (Figure 8). Ten *MtWAK-RLKs* transcripts were induced after 2 h of drought (Figure 8A). Seven and four *MtWAK-RLKs* were up-regulated or down-regulated by cold stress, respectively (Figure 8B), and three and four *MtWAK-RLKs* were up-regulated or down-regulated by salt stress, respectively (Figure 8C). The results indicate that *MtWAK* expression is probably involved in abiotic stress resistance.

## 4. Materials and Methods

### 4.1. Identification of MtWAK Genes

The genome sequences were obtained from *M. truncatula* genome database (http://www.medicagogenome.org/, Mt4.0v2). The gene information of *WAK* family in *Arabidopsis* and Rice were retrieved from the previous studies. The hidden Markov model (HMM) profiles of the WAKs were down-loaded from the Pfam database (http://pfam.xfam.org/, accessed on 1 August 2020). EGF_CA(PF07645), WAK_assoc (PF14380), GUB_WAK_bind (PF13947) and Pkinase_Tyr (PF07714) were used to identify MtWAKs. Firstly, BLASTP search was performed at *M. truncatula* genome database with an *e*-value of 1e-5 using previously reported sequences of AtWAKs and OsWAKs as query. Then, we searched EGF_CA(PF07645), WAK_assoc (PF14380), GUB_WAK_bind (PF13947) and Pkinase_Tyr (PF07714) domain from putative MtWAKs with e-value cut-off at 1.0 by using HMMER v3.1b2 software. The integrity of the four domains was verified by using the online program SMART (http://smart.emblheidelberg.de/, accessed on 1 August 2020) with an e-value < 0.1. Lastly, each candidate gene was assessed for its sequence similarity to other putative MtWAKs. Only genes that fit into one of the four *MtWAK* types (see Table S1) according to Zhang et al. [18] were defined as *MtWAKs*. Protein length, molecular weight (MW) and isoelectric point (PI) were predicted by ExPasy program (http://www.expasy.org/tools/, accessed on 10 August 2021).

### 4.2. Chromosomal Location and Synteny Correlation Analysis

The physical position of the *MtWAK* genes on the chromosome was mapped using TBtools software (Version 1.108). The synteny correlation analysis of *WAK* genes between the homologs in *M. truncatula* and *A. thaliana* or *G. max* were verified and visualized using TBtools software.

### 4.3. Phylogenetic Analysis and Gene Structure, Motif and Conserved Domain Analysis 

Multiple alignments of protein sequences were performed using CLUSTALX software (Version 1.81). The phylogenetic tree was constructed by using MEGAX with the neighbor-joining method and 1000 bootstrap replications. The gene structure, conserved domain and conserved motifs were displayed using TBtools software. The conserved domains and conserved motifs of the 26 *MtWAK-RLKs* were analyzed by SMART program and MEME program (Version 5.4.1) (http://meme-suite.org/tools/meme, accessed on 20 July 2022).

### 4.4. Analysis of cis-Acting Regulatory Elements

The 2000 bp promoter sequences upstream from the initiation codon of *MtWAK-RLKs* were extracted from the genome of *M. truncatula* and analyzed using PlantCARE software (http://bioinformatics.psb.ugent.be/webtools/plantcare/html/, accessed on 1 August 2020). However, less than 300 bp promoter sequence of *MtWAK10* was available.

### 4.5. Expression Pattern Analysis

The genome-wide microarray data obtained from MtGEA (https://mtgea.noble.org/v3/, accessed on 1 August 2020) was used to analyze the expression of *MtWAK* genes in different tissues and developing stages. The expression data were gene-wise normalized. The clustered heatmap of expression pattern profile on log2 scale was portrayed using TBtools software. Analyses of *WAK* expression in response to biotic and abiotic stress were conducted on datasets: (ⅰ) ‘Cell suspension_Yeast elicitor’, ‘Root_*Macrophomina* infected’ and ‘Root Tip_A17_*Ralstonia*’ (microarray data obtained from MtGEA); (ⅱ) powdery mildew *Erysiphe pisi* treatment [48] RNA-seq data were retrieved from NCBI Database (SRR7589436, SRR7589435, SRR7589438, and SRR7589437); (ⅲ) drought, salt and cold treatment, date from NCBI GEO (dataset accession: GSE136739). The expression abundance was presented by the reads per kilobase per million (Table S3). The relative transcript level after treatments was calculated compared with the untreated control or before treatment (0 h). The clustered heatmap of relative expression pattern on log2 scale was analyzed by the TBtools.

### 4.6. Tissue Samples Collection and Cold and Salt Treatment

*M. truncatula* plants were grown in a growth chamber at 25 °C with 16 h of light. Root, stem, mature leave, flower, pod and seed were sampled from three-month-old plants. Six-week-old plants were exposed to low temperature (5 °C) as a cold stress treatment. Leaves were collected at time intervals of 0, 1, 2, 6 and 12 h.

### 4.7. RNA Extraction and qRT-PCR

Total RNA was extracted using the RNAprep pure Plant Kit (Tiangen, Beijing, China). cDNA synthesis was performed with HiScript III RT SuperMix for qPCR (+gDNA wiper) reagent kit with gDNA Eraser (Vazyme, Nanjing, China). qRT-PCR was performed following the instructions of ChamQ Universal SYBR qPCR Master Mix (Vazyme, Nanjing, China). The *MtActin7* (*Medtr3g095530*) gene was used as the controls. All primer sequences are shown in Supplementary Table S5. The relative gene expression level was calculated with 2^−Δ*Ct*^ method.

### 4.8. Subcellular Localization

The full CDSs of *MtWAK24* without the terminator were cloned and ligated to pCAMBIA1305-GFP vector driven by *CaMV 35S* promoter. Primer sequences for constructions are shown in Supplementary Table S5. Positive Agrobacterium colonies were cultured in LB medium containing 50 μg/mL rifampicin and kanamycin at 28 °C overnight; the cells were harvested by centrifugation at 2500 × *g* at room temperature for 3–5 min and resuspended in infiltration buffer (10 mM MgCl_2_, 10 mM MES pH 5.6, and 100–200 µM acetosyringone). The cell density was adjusted to OD_600_ = 0.2 before the cell suspensions were infiltrated into one-month-old *Nicotiana benthamiana* (*N. benthamiana*) leaves. After 48–72 h, fluorescence was observed using confocal laser scanning microscopy (Zeiss LSM800, Jena, Germany).

### 4.9. Phytophthora Infection in N. benthamiana

Vectors expressing selected *MtWAK-RLKs* under control of *CaMV 35S* promoter were constructed. The infection experiments were performed as described [49]. Briefly, the infiltrated leaves were inoculated with *P. parasitica* mycelium at 12 hpi, and leaf lesions were determined using the Evans blue method at 48 hpi. *P. parasitica* strain was obtained from Yuanchao Wang‘s lab (Nanjing Agricultural University, Nanjing, China). Relative biomass of *P. parasitica* was determined by qPCR of *P. parasitica* genome DNA normalized to tobacco genome DNA at 48 hpi. All primer sequences are shown in Supplementary Table S5.

## 5. Conclusions

In summary, a comprehensive genome-wide analysis of *WAK* family was performed. A total of 54 *MtWAKs* were identified in *M. truncatula*, including 26 *MtWAK-RLK*, 9 *MtWAK-RLCK*, 10 *MtWAK-RLP* and 9 short protein type genes. *MtRLKs* and *MtRLCKs* were largely tandem duplicated. Most *MtWAK-RLKs* had similar exon–intron organization and motif distribution. Multiple *cis*-acting elements responsible for phytohormones, stresses, growth and development were observed in the promoter regions. The expression patterns of *MtWAK-RLKs* varied in different plant tissues and developmental stages and biotic and abiotic stress conditions. The results suggest that *MtWAKs* might have multiple functions in *M. truncatula.* Furthermore, plasma-membrane-localized MtWAK24 significantly inhibited *Phytophthora* infection in tobacco, indicating its role in pathogen resistance, which is worthy to be investigated in the future.

## Data Availability

The data that support the findings of this study are available from the corresponding author upon reasonable request.

## Supplementary Materials

The following supporting information can be downloaded at: www.mdpi.com/article/10.3390/plants12091849/s1, Figure S1: The motif of MtWAK-RLKs; Table S1: The predicated information of *MtWAK* family genes in *M. truncatula*. Table S2: The synteny analysis of *WAK* homologs between *M. truncatula* and *A. thaliana* or *G. max*; Table S3: The gene expression profiles of *MtWAK-RLKs* in different tissues and developmental stages as well as biotic and abiotic stress; Table S4: The number of plant protein kinases and WAKs predicted via iTAK program; Table S5: Primers.
