# Supplementary figures and images for: Genome-Wide Analysis of the Wall-Associated Kinase (WAK) Genes in Medicago truncatula and Functional Characterization of MtWAK24 in Response to Pathogen Infection
Source: Plants (Basel). 2023 Apr 30;12(9):1849. doi: 10.3390/plants12091849 (PMC10180995; doi:10.3390/plants12091849)

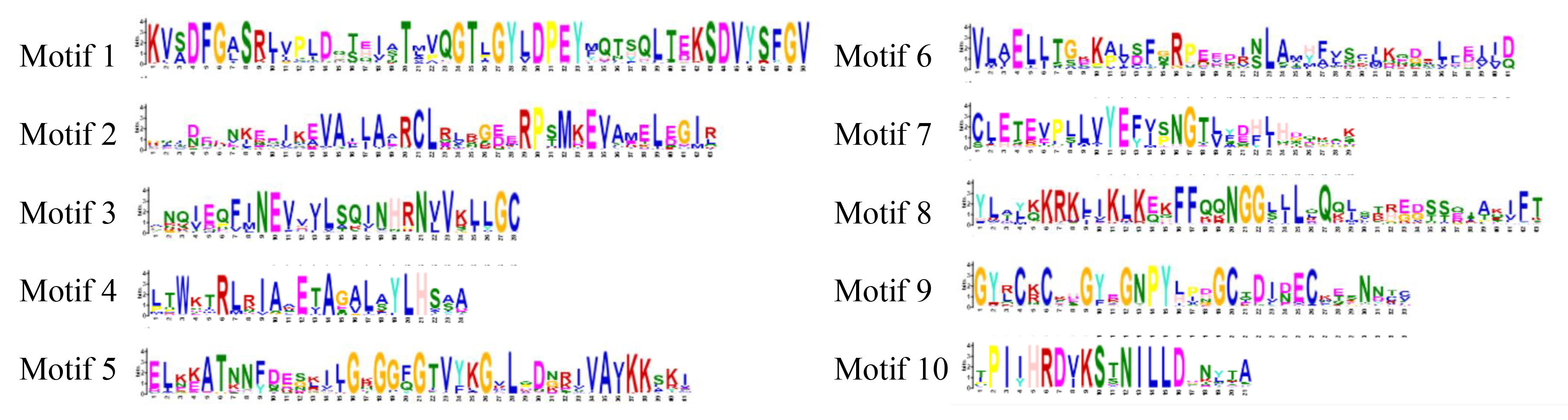

Supplement: Supplementary file 1 [file plants-12-01849-s001.zip › figure S1.jpg]
